# Estimation of Gestational Age, Using Neonatal Anthropometry: A Cross-sectional Study in India

**DOI:** 10.3329/jhpn.v31i4.20051

**Published:** 2013-12

**Authors:** Rajat Thawani, Pooja Dewan, M.M.A. Faridi, Shilpa Khanna Arora, Rajeev Kumar

**Affiliations:** University College of Medical Sciences, Delhi, India

**Keywords:** Anthropometry, Equation, Gestational age, New Ballard Score, Newborn, Indias

## Abstract

Prematurity is a significant contributor to neonatal mortality in India. Conventionally, assessment of gestational age of newborns is based on New Ballard Technique, for which a paediatric specialist is needed. Anthropometry of the newborn, especially birthweight, has been used in the past to predict the gestational age of the neonate in peripheral health facilities where a trained paediatrician is often not available. We aimed to determine if neonatal anthropometric parameters, viz. birthweight, crown heel-length, head-circumference, mid-upper arm-circumference, lower segment-length, foot-length, umbilical nipple distance, calf-circumference, intermammary distance, and hand-length, can reliably predict the gestational age. The study also aimed to derive an equation for the same. We also assessed if these neonatal anthropometric parameters had a better prediction of gestational age when used in combination compared to individual parameters. We evaluated 1,000 newborns in a cross-sectional study conducted in Guru Teg Bahadur Hospital in Delhi. Detailed anthropometric estimation of the neonates was done within 48 hours after birth, using standard techniques. Gestational age was estimated using New Ballard Scoring. Out of 1,250 consecutive neonates, 1,000 were included in the study. Of them, 800 randomly-selected newborns were used in devising the model, and the remaining 200 newborns were used in validating the final model. Quadratic regression analysis using stepwise selection was used in building the predictive model. Birthweight (R=0.72), head-circumference (R=0.60), and mid-upper arm-circumference (R=0.67) were found highly correlated with gestation. The final equation to assess gestational age was as follows: Gestational age (weeks)=5.437×W–0.781×W^2^+2.815×HC–0.041×HC^2^+0.285×MUAC–22.745 where W=Weight, HC=Head-circumference and MUAC=Mid-upper arm-circumference; Adjusted R=0.76. On validation, the predictability of this equation is 46% (±1 week), 75.5% (+2 weeks), and 91.5% (+3 weeks). This mathematical model may be used in identifying preterm neonates.

## INTRODUCTION

Neonatal survival has improved worldwide, albeit at a slow pace. This is especially true for developing countries which still account for almost all neonatal deaths (99%) in the world (1). Amongst the 193 member states of WHO, for whom the statistics for neonatal deaths are available, India has the highest number of annual neonatal deaths. Out of the 3.072 million neonatal deaths reported worldwide by the World Health Organization (WHO) in 2010, nearly one-third (875,000) occurred in India (2). India, Nigeria, Pakistan, China, and Congo together account for more than 50% of all neonatal deaths globally.

During the past two decades, there has been a sustained reduction in infant and child mortality rate but the reduction in neonatal mortality rate (NMR) is far from satisfactory (1,3). The contribution of newborn deaths to the under-5 mortality has grown from 37% in 1990 to 41% in 2011 (4). To bring about a decrease in NMR, there is a need to curtail the three most important causes of neonatal deaths, *viz*. preterm delivery (29%), asphyxia (23%), and severe infections, such as sepsis and pneumonia (25%). An estimated 1 million babies die globally every year because of prematurity, of which about 375,000 neonatal deaths due to prematurity and low birthweight occur in India alone (5,6).

Conventionally, gestational age of neonates is computed based on Naegele's formula or by ultrasonic evaluation during pregnancy, or after birth, using New Ballard assessment and scoring (6). Gestational age estimates based on Naegele's formula have lower accuracy in settings with low literacy (7) and are likely to be affected by variation in ovulation and also by breastfeeding. Ultrasound, as a tool to assess gestational age, is a limiting factor, particularly in developing countries, like India where only 51% of women undergo the recommended number of at least 3 antenatal visits; 59% of deliveries take place at home; and only 24% of pregnant women undergo ultrasonic evaluation during pregnancy (8). Assessment of gestational age of newborns using New Ballard Score (NBS) may not be reliable as its accuracy depends on the skill of examiner (9) and the condition of the neonate. It cannot be used in asphyxiated neonates. In addition, it is a complex score, which requires the skills of a paediatric specialist. Thus, there is need to develop a simple, inexpensive and practical method to identify these highly-vulnerable preterm newborns soon after birth (10,11).

We conducted this study to devise a mathematical model to predict the gestational age of a neonate, using anthropometric estimates, like crown heel-length, upper segment-length, lower segment-length, head-circumference, mid-upper arm-circumference, foot-length, intermammary distance, and umbilical nipple distance, using these parameters alone or in combination.

## MATERIALS AND METHODS

This cross-sectional study was conducted in the neonatology division of University College of Medical Sciences and GTB Hospital, a tertiary hospital in Delhi, India. We assessed consecutive singleton neonates and their mothers within 48 hours after birth between July 2011 and December 2011 for possible inclusion in the study. Neonates for whom reliable information about gestational age was not available (mother not aware of the beginning of her last menstrual period; irregular menstrual cycles prior to pregnancy; bleeding during the first two months of pregnancy; use of oral contraceptives before pregnancy, lactational amenorrhoea) and those with gross congenital anomalies and severe birth asphyxia were excluded from the study. Gestational age of the newborns was calculated from the case sheets of the mother, using Naegele's formula (12) and by NBS (13) which was regarded as the gold standard for our study. A detailed anthropometric assessment was performed for each of the subjects between 24 and 48 hours after birth. To avoid inter-observer bias, the anthropometric estimations and the assessment of gestational age by NBS were carried out by only one of the investigators.

The baby was weighed in nude, using a digital electronic scale (Goldtech, Merino International) to the nearest 5 g. The recumbent crown heel-length (CHL) was recorded using an infantometer (Seca) to the nearest 1.0 mm. The mid-upper arm-circumference (MUAC) was measured at the midpoint between the tip of the acromion and olecranon process of the left upper arm. The head-circumference (HC) was measured between the glabella anteriorly and along the most prominent point posteriorly by cross-over technique, measured over the parietal eminence. The lower segment (LS) was measured as the distance between the pubic symphysis to the heel. The umbilical nipple distance (UND) was measured between the 12 o'clock position of the rim of the umbilicus to the right nipple. The calf-circumference (CaC) was measured at the most prominent point in a semi-flexed position of the leg. MUAC, HC, LS, UND, and CaC were measured using a non-stretchable cloth-measuring tape to the nearest 1.0 mm. The foot-length (FL) was measured as the distance from the heel to the longest toe of the right foot, parallel to the long axis of the foot, using the paddle blades of an automated slide calliper. The hand-length (HL) was also used by measuring the distance between the heel of the hand and tip of the middle finger, with the wrist held in extension and the palm and fingers extended against the hand of the assistant, using a slide calliper. The intermammary distance (IMD) was measured at the end of expiration, using callipers (14). All the measurements were done 3 times, and the mean value was used in analysis. All anthropometric parameters were recorded in a predesigned proforma. Neonates were categorized as ‘small’, ‘large’, and ‘appropriate’ for gestational age, using Lubchenco's reference charts (15).

All study subjects were recruited after obtaining informed written consent from the parents/guardians. Approval was also obtained from the Institutional Ethics Committee of Guru Teg Bahadur Hospital.

### Statistical methods

Using the data of randomly-selected 800 neonates out of 1,000, the relationship between each of the 10 anthropometric indicators (W, CHL, HC, MUAC, US, CaC, UND, HL, FL, and IMD) and the gestational age as determined by the New Ballard Score was derived, using linear and non-linear regression. We derived regression equations for each anthropometric predictor, with the gestational age of the newborn according to the New Ballard Score. Pearson's correlation was used in detecting collinearrity between any two anthropometrical predictors. If the correlation between the predictors exceeded 0.8, these were considered to have a high collinearity.

Anthropometric variable with the maximum R was selected to derive an equation to predict gestational age, using backward elimination. Subsequently, the remaining anthropometric variables with lesser R were added to the above equation in a stepwise manner. If the change in the adjusted R square was more than 0.005 by including an anthropometric predictor in the equation, the predictor was retained in the equation, else it was rejected. The final model was checked for normality of residuals and outliers.

The equation so derived was then validated on a set of 200 neonates randomly selected from the total population, using a computer-generated random number table.

## RESULTS

We assessed 1,250 consecutive singleton neonates delivered in the hospital and their mothers for possible inclusion in the study. The babies for whom reliable information about gestational age was not available [mother not aware of the beginning of her last menstrual period (n=88); irregular menstrual cycles prior to pregnancy (n=82); bleeding during the first two months of pregnancy (n=14); use of oral contraceptives before pregnancy (n=26)] were excluded. Babies with gross congenital anomalies (n=6), severe birth asphyxia (n=30), and those born to mothers with lactational amenorrhoea (n=4) were also excluded from the study. A total of 1,000 neonates, ranging in weight from 685 to 4,165 g (mean±SD=2,395+597 g) were recruited within 48 hours after their birth between July 2011 and December 2011. Their gestational age varied from 25 to 42 weeks, with 373 neonates (37.3%) being preterm and 62.7% being term. Only 1.3% of neonates were found to be extremely premature (<28 weeks gestation). Eight hundred and fifty neonates were found appropriate for gestational age (AGA); 142 neonates were small-for-gestational age (SGA); and 8 neonates were large-for-gestational age (LGA) when classified according to Lubchenco charts (15). Table 1 depicts the baseline characteristics of neonates recruited in our study. Almost all the study subjects hailed from urban slums of northeast national capital territory region of Delhi and belonged to lower socioeconomic stratum of the society.

**Table 1.** Baseline characteristics of neonates included in the study (n=1,000)

Gestational age* (weeks)	Gestational profile			No. of neonates	Number of males	Birthweight (kg)**	Mid-upper arm- circumference (cm)**	Head- circumference (cm)**
	AGA (n)	SGA (n)	LGA (n)					
24-<26	5	0	0	5	2	0.99 (±0.13) [0.84-1.21]	6.38 (+0.62) [5.5-7.1]	25.44 (+0.91) [24.5-26.6]
26-<28	8	2	0	10	3	1.04 (±0.30) [0.75-1.7]	5.87 (+0.37) [5.4-6.4]	27.44 (+2.02) [23.4-30.1]
28-<30	12	2	0	14	7	1.12 (+0.26) [0.68-1.57]	6.15 (+0.53) [5.2-7.3]	25.66 (+2.19) [23.0-30.6]
30-<32	53	0	3	56	25	1.51 (+0.33) [1.1-2.4]	7.50 (+1.11) [5.0-10.3]	30.20 (+2.02) [26.0-34.0]
32-<34	65	25	1	91	31	1.70 (+0.33) [1.3-3.8]	8.06 (+0.95) [6.0-9.5]	30.61 (+1.73) [26.2-35.0]
34-<36	215	30	0	245	113	2.31 (+0.37) [1.3-3.8]	9.44 (+1.18) [6.3-12.0]	33.04 (+2.12) [28.0-40.0]
36-<38	300	55	4	388	164	2.61 (+0.56) [1.25-4.17]	9.97 (+1.48) [6.8-14.0]	33.53 (+1.82) [25.0-39.0]
38-<40	152	27	0	179	65	2.82 (+0.59) [1.2-3.8]	10.34 (+1.22) [7.0-13.0]	34.17 (+1.62) [31.0-40.0]
40-42	40	1	0	41	23	3.05 (+0.69) [2.6-3.5]	10.04 (+0.87) [8.4-11.2]	34.8 (+1.27) [32.4-35.8]

*By Modified Ballard Criteria;

**Values expressed as means (±SD);

AGA=Appropriate-for-gestational age;

LGA=Large-for-gestational age;

SGA=Small-for-gestational age;

Figures in square parenthesis indicate range

Table 2 depicts the correlation between various anthropometric predictors, and none of the predictors had bivariate correlation more than 0.8. The relationship between each of the 10 anthropometric predictors and the gestational age of the newborn as determined by NBS is shown in Table 3. Since, some of the anthropometric measurements had a significant non-linearity, the quadratic equations were preferred. Four anthropometric measurements had a better quadratic correlation with gestational age, viz. weight, mid-upper arm-circumference, upper segment-length, and head-circumference. The equations (linear and quadratic) for determining gestational age are shown in Table 3. Birthweight had the best correlation with GA (R=0.72), and addition of HC, HC^2^, and MUAC improved the correlation significantly (R=0.76). While computing the final equation, we included all significant predictors using ‘backward elimination method’ with change in p>0.10 to remove the other predictors; we retained W, W^2^, HC, HC^2^, and MUAC in the final equation. Subsequently, all other anthropometric predictors and their quadratic terms were added one by one in a stepwise manner (Table 4). Since inclusion of none of the other predictors caused an increase in R^2^ by >0.005, these were not retained in the final equation mentioned earlier.

### Validation

The equation was validated on 200 newborns picked randomly from the 1,000 neonates (preterm 48%, term 52%). The predictability of the equation was 91.5% when the predicted gestational age was found to be within 3 weeks of that determined clinically by NBS. Likewise, the prediction was 75.5% and 46% within 2 and 1 week(s) respectively. The figure shows the relationship between the predicted gestational age and assessed gestational age (by New Ballard Score). The overall correlation between the predicted gestational age and that determined by NBS was good (R=0.89, R2=0.79). In preterm neonates (<37 weeks gestation), the correlation coefficient was 0.84 while, in term neonates, it was 0.44.

Using NBS, the preterm neonates were 324 in number but, by using the equation, there were 419 preterm neonates. The equation had a sensitivity of 79%, specificity of 65.5%, positive predictive value of 61.1%, and negative predictive value of 82%, for detecting preterm neonates.

**Table 2.** Pearson's correlation (R) between different anthropometric predictors

	BW	CHL	HC	MUAC	US	IMD	UND	FL	HL	CaC
BW	1	0.704**	0.625**	0.757**	0.560**	0.584**	0.593**	0.508**	0.501**	0.714**
CHL	0.704**	1	0.605**	0.696**	0.619**	0.583**	0.563**	0.432**	0.400**	0.598**
HC	0.625**	0.605**	1	0.607**	0.450**	0.526**	0.501**	0.440**	0.331**	0.526**
MUAC	0.757**	0.696**	0.607**	1	0.540**	0.589**	0.603**	0.499**	0.460**	0.720**
US	0.560**	0.619**	0.450**	0.540**	1	0.431**	0.486**	0.389**	0.366**	0.510**
IMD	0.584**	0.583**	0.526**	0.589**	0.431**	1	0.679**	0.506**	0.515**	0.595**
UND	0.593**	0.563**	0.501**	0.603**	0.486**	0.679**	1	0.431**	0.412**	0.631**
FL	0.508**	0.432**	0.440**	0.499**	0.389**	0.506**	0.431**	1	0.591**	0.486**
HL	0.501**	0.400**	0.331**	0.460**	0.366**	0.515**	0.412**	0.591**	1	0.433**
CaC	0.714**	0.598**	0.526**	0.720**	0.510**	0.595**	0.631**	0.486**	0.433**	1

**Correlation is significant at 0.01 level (2-tailed);

BW=Birthweight;

CaC=Calf-circumference;

CHL=Crown heel-length;

FL=Foot-length;

HC=Head-circumference;

HL=Hand-length;

IMD=Intermammary distance;

MUAC=Mid-upper arm-circumference;

UND=Umbilical nipple distance;

US=Upper segment

**Table 3.** Linear and quadratic regression equations for estimating gestational age (in weeks) based on anthropometry of neonates

Anthropometric predictor	Linear regression equation [R]	Quadratic (best non-linear) regression equation [R]
Weight (W)	GA=30.23+(2.57×W) [0.67]	GA=24.07+(8.15×W)-(1.19×W^2^) [0.72]
Crown heel-length (CHL)	GA=20.06+(0.34×CHL) [0.56]	GA=-8.40+(1.57×CHL)-(0.01×CHL^2^) [0.58]
Head-circumference (HC)	GA=19.73+(0.504×HC) [0.52]	GA=-67.74+(5.82×HC)-(0.08×HC^2^) [0.60]
Mid-upper arm- circumference (MUAC)	GA=26.40+(1.03×MUAC) [0.64]	GA=13.59+(3.78×MUAC)-(0.15×MUAC^2^) [0.67]
Upper segment (US)	GA=28.61+(0.27×US) [0.20]	GA=25.88+(0.470×US)-(0.004×US^2^) [0.60]
Calf-circumference (CaC)	GA=26.33+(0.99×CaC) [0.45]	GA=17.36+(2.85×CaC)-(0.09×CaC^2^) [0.56]
Umbilical nipple distance (UND)	GA=27.54+(0.867×UND) [0.48]	GA=5.11+5.31×UND)-(0.22×UND^2^) [0.52]
Hand-length (HL)	GA=28.76+(1.28×HL) [0.40]	GA=4.80+(9.26×HL)-(0.66×HL^2^) [0.47]
Foot-length (FL)	GA=27.45+(1.20×FL) [0.43]	GA=-8.237+(10.69×FL)-(0.62×FL^2^) [0.53]
Intermammary distance (IMD)	GA=28.85+(0.94×IMD) [0.48]	GA=13.19+(4.87×IMD)-(0.24×IMD^2^) [0.53]

**Table 4.** Deriving the final equation by forced entry into the equation

Predictor included by forced entry one by one	R (0.764)*	R^2^ (0.583)*	Adjusted R^2^ (0.581)*	Change in adjusted R^2^
CHL and CHL^2^	0.764	0.584	0.580	-0.001
IMD and IMD^2^	0.766	0.587	0.583	+0.002
UND and UND^2^	0.765	0.585	0.581	-
FL and FL^2^	0.765	0.586	0.582	+0.001
HL and HL^2^	0.764	0.584	0.580	-0.001
CaC and CaC^2^	0.765	0.586	0.582	+0.001
US and US^2^	0.767	0.588	0.584	+0.003

*Values mentioned in parenthesis indicate those for the equation Gestational age=5.437 W-0.781 W^2^+2.815 HC–0.041 HC^2^+0.285 MUAC–22.745

## DISCUSSION

Prematurity is a major determinant of neonatal survival. With increasing gestational age, neonatal survival rate increases to 15% at 23 weeks, 56% at 24 weeks, and 79% at 25 weeks (16). Estimation of gestational age by methods, like ultrasonic assessment and recall of last menstrual period, is prone to error and difficult to use in resource-poor countries. In developing countries, less than half of the neonates undergo any evaluation within 24 hours of birth; In India, only 41% of neonates undergo any check-up within 24 hours after birth (17). According to the estimates of preterm births in 2005, about 9.6% of all births were preterm. About 12.9 million births worldwide were definable as preterm. Approximately 85% of this burden was concentrated in Africa and Asia where 10.9 million births were preterm. About 0.5 million preterm births occurred in Europe and the same number in North America while 0.9 million occurred in Latin America and the Caribbean (18). Given these estimates, a sizeable number of preterm births can be identified using this formula; 75% of neonates would be identified with an accuracy of ±14 days. On validation, our results were found to have greater merit in assessing preterm neonates.

We found a good linear correlation between gestational age and birthweight, crown heel-length, mid-upper arm-circumference, and head-circumference. The quadratic correlation coefficients for birthweight, head-circumference, and mid-upper arm-circumference were the highest and, hence, included in the final equation. The quadratic term for MUAC was not retained in the final equation as its inclusion did not result in any significant increase in R (p=0.91). Likewise, by including US and US^2^, we did not find any significant increase in R and would have only made the equation more complex. MUAC has been previously found to have significant correlation with gestational age in neonates (19-21). Sasanow *et al*. (19) found a significant (p<0.001) linear correlation between HC (R=0.95), MUAC (R=0.93), and MUAC/HC (R=0.84) with the estimated gestational age between 25 and 42 weeks. Excler et *al*. (21) also found a significant (p<0.001) linear correlation between MUAC and gestational age in AGA (R=0.850) as well as SGA (R=0.76) neonates. Excler *et al*. (21) also established an equation to estimate gestational age, using mid-upper arm-circumference and subscapular skinfold thickness taken exactly 15 second after application of the calliper (SSKF_15_) and chest-circumference (ChC) after multiple regression analysis: Gestational age (weeks)=1.216 MUAC (cm)-3.588 SSKF_15_ (mm)+0.263 ChC (cm)+17.9. In our equation, W and W^2^ alone could explain 54.2% variation in predicted GA, and addition of HC, HC^2^, and MUAC could account for an additional 4% variation in GA, which was statistically significant.

**Figure.**
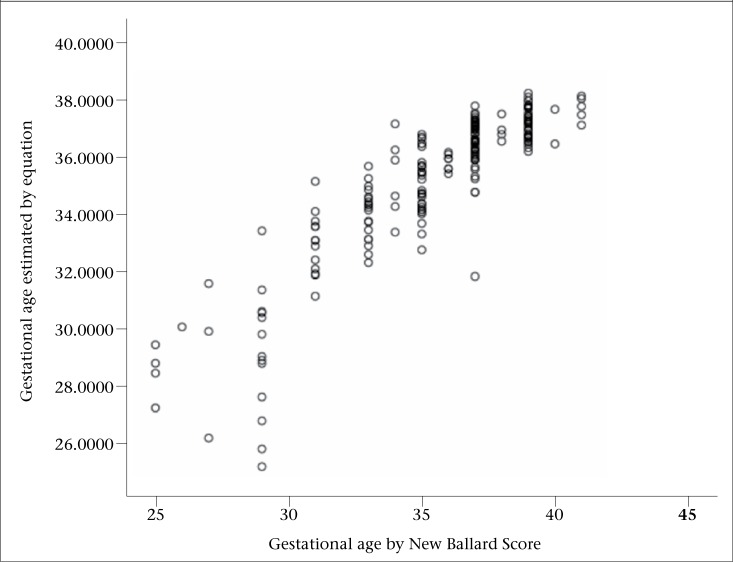
Correlation between predicted gestational age (using equation) and gestational age assessed by New Ballard Score (n=200)

Despite being trained in external Ballard examination (a form of clinical examination) to assess gestational age, experienced health workers showed poor skill development (22). In contrast, anthropometric measurements collected by health workers have shown to be more reliable than clinical examination (23,24). In addition, long-term retention of knowledge and skills of primary health workers in managing sick children have been low. Hence, providing a ready mathematical formula to assess gestational age may be a viable option for community health workers (25).

### Strengths and limitations of the study

The main limitation of our study was that the equation was derived and validated in an institutional set-up. However, the strengths of our study include a large sample. The predictors used in our equation are simple and can be recorded even by unskilled personnel. In addition, our results were found to have a greater validity in identifying the preterm neonates, thereby justifying our primary aim.

### Conclusions

Since our results showed that the variation in GA determined using this equation and that by NBS is 3 weeks, the equation should be used cautiously and as an alternative to NBS in settings where antenatal ultrasound and skilled clinicians to assess the neonate's gestational age within 72 hours after birth are not available. We also recommend a similar study in the community.
